# Patients’ anxiety around incidental brain tumors: a qualitative study

**DOI:** 10.1007/s00701-013-1935-2

**Published:** 2013-11-24

**Authors:** Harshita Jagadeesh, Mark Bernstein

**Affiliations:** Division of Neurosurgery, Toronto Western Hospital, 399 Bathurst St., Toronto, ON M5T 2S8 Canada

**Keywords:** Incidental findings, Brain MRI, Neurosurgery, Qualitative, Worrisome news

## Abstract

**Background:**

Incidental findings are common on MRI. Our study examined how patients are told about their incidental finding as well as anxiety until the neurosurgical consultation and afterward.

**Methods:**

Qualitative research methodology was used. Thirty-two participants were interviewed using open-ended questions. Answers were transcribed and analyzed for themes.

**Results:**

The level of patient satisfaction for the initial breaking of the news averaged 4.1 (range 1–5). Four themes were identified: (1) emotional stress over incidental findings are partially dependent on how the news was communicated; (2) breaking worrisome news is best done in person, but telephone communication can sometimes be acceptable; (3) patients are divided about how much information they wish to get about incidental findings before going for an MRI; (4) waiting for the neurosurgical consultation is a stressful time without adequate support.

**Conclusions:**

When dealing with an unexpected MRI finding, patients are anxious about the situation. Our study exposes ways the experience could be made more comfortable for patients right from the start, from being told the news in a calm and sympathetic manner, to providing support for patients while they wait for a meeting with a neurosurgeon, to expediting the neurosurgical consultation.

Incidental findings on brain MRIs are common in the general population [8], occurring in about 5–20 % of all examinations [2]. The prevalence of these findings is increasing with more routine use of MRI and is also directly proportional to age [8, 15]. In a review of 16 publications with 20,000 participants, the incidence of incidental tumors was 0.7 % [8]. In another study, 4.4 % of young, healthy volunteers had an incidental brain finding [10]. A recent study found that the most common reason to obtain an MRI scan in children was in the setting of an accidental head injury [11], and in all cases the incidental finding bore no relationship with the patient’s head injury.

However, the chance of finding a clinically relevant result is rare, about 0.3 to 3.4 % in the general population [2]. It is less than 0.1 % in the case of finding a malignant brain tumor [15]. In children who presented with headaches, which led to an incidental finding on the resulting scan, only about 17 % of those had to undergo surgery [11]. Most findings are not urgent; only about 1–2 % need immediate referral to a specialist [13], but all findings still need to be followed up by a neurosurgeon. In these cases, the primary physician must first break the news to the patient who must then wait for a consultation before finding out more definitive information. This can be a daunting and scary time [8].

Due to the possibility of an incidental finding, some have proposed that patients and research volunteers should be made aware of this possibility when sent for an MRI [8, 11]. However, little has been done to put the necessary standards in place, as there is a general lack of knowledge regarding how patients would like to be informed of the news and how to best handle the situation by the specialist [10].

There are a few articles on incidental brain tumor findings in adults [8]. Several studies have looked at how to break the news initially, from the patient’s point of view, the physician’s, and the intern’s in a hospital [3, 7, 9, 14]. The present qualitative study looked specifically at patients with incidental MRI findings focusing on patients’ feelings and anxiety after the initial knowledge of their incidental finding and during the time leading up to a consultation with the neurosurgeon. Interviews were conducted face to face, with open-ended questions and case scenarios. The answers were transcribed and analyzed for themes and recurring ideas.

The intention of this study is to help provide a better understanding of how to proceed with the discovery of an incidental finding and the best way, from the patients’ perspective, to seamlessly deliver the news and expedite a neurosurgical consultation so as to minimize the fright for the patient and to make the process more comfortable.

## Patients and methods

### Study design

This was a qualitative study conducted using one-on-one interviews at Toronto Western Hospital, a tertiary and quaternary referral neurosurgical center. Interviews used open-ended semi-structured questions and case scenarios (Appendix 1).

### Participants

All patients interviewed were >18 and had come for a neurosurgical consultation with the principle investigator (MB) regarding an incidental finding on their MRI scan. Informed consent was obtained from all of the participants, and the goals of the study were explained prior to participation. Participants were excluded if communication in English was poor and/or appropriate translation was not possible, if they were emotionally unprepared for the interview, or if they were not cognitively intact.

### Sample size

During the course of the study, approximately 30 participants were sought, using the method of convenience sampling. This number complies with the usual sample size necessary for significance of qualitative data and for saturation of themes to be reached [6].

### Data collection

Data were collected in the form of transcriptions of patients’ answers to the open-ended questions and case scenarios. Additional questions were added to the interview guide as more common themes started to be noticed among the patient responses.

Demographic data were obtained for each participant including age, martial status, highest education level, the time frame from when patients first found out about the incidental finding until the consultation with the neurosurgeon, level of satisfaction, and their diagnosis (Table 1).

**Table 1:** Diagnostic Information for All Participants

ID no.	Age	Sex	Marital status	Highest education level	Occupation	Time taken from symptoms to FP report (weeks)	Method of communication	Level of satisfaction	Diagnosis
1	50	F	Married	University BA	Insurance broker	16	In person	5	Benign
2	50	F	Single	University BSC	Unemployed	4	Telephone	1	Benign
3	60	F	Divorced	University diploma	Marketing	24	In person	5	Benign
4	57	F	Divorced	Masters clinical science	Speech language pathologist	12	In person	4.5	Benign
5	75	F	Married	Highschool diploma	Retired	4	In person	5	Benign
6	42	F	Seperated	University BA	Revenue manager	24	Telephone	3	Benign
7	57	F	Divorced	Highschool diploma	Self-employed	4	In person	5	Benign
8	27	M	Single	University BA	Student	1	In person	5	Benign
9	41	F	Married	Highschool diploma	Self-employed	12	In person	4	Benign
10	36	F	Married	Highschool diploma	CIBC teller	8	In person	4	Benign
11	54	F	Married	University BA	Unemployed	4	In person	4.5	Benign
12	42	F	Married	College degree	Admin support	24	In person	3	Benign
13	42	M	Common-law	Masters	Health care admin director	5	In person	5	Cavernoma
14	76	F	Married	Highschool diploma	Retired	24	In person	5	Benign
15	65	M	Married	University diploma	Retired	4	In person	5	Benign
16	22	F	Single	Highschool diploma	Student	1	In person	2.5	Low-grade giloma
17	62	M	Married	Engineering degree	Security officer	12	In person	5	Blood clot in brain
18	54	F	Common-law	College degree	Self-employed	3	Telephone	5	Benign
19	65	F	Married	Grade 8	Seamstress	3	Telephone	5	Benign
20	44	F	Married	Masters	Registered nurse	1	In person	5	Benign
21	70	F	Single	Grade 6	Sales clerk	24	In person	5	Benign
22	54	F	Divorced	Highschool diploma	Executive assisstant	24	Telephone	5	Benign
23	68	M	Married	Engineering degree	Retired	36	In person	5	Benign
24	28	M	Single	College degree	Graphic designer	8	In person	4	Benign
25	44	F	Married	University BA	Self-employed	12	In person	3	Benign
26	49	M		College	Chef	1	In person	5	Benign
27	43	F	Married	Grade 12	Medical office admin	3	In person	2	Benign
28	18	F	Single	Highschool diploma	Student	24	In person	5	Benign
29	61	F	Married	Grade 7	Housewife	4	In person	3	Cavernoma
30	49	F	Married	University BSC	Accounting assisstant	4	In person	4	Benign
31	75	M	Married	Masters	Retired	1	In person	5	Benign
32	56	F	Divorced	College	Law clerk	8	In person	1	Benign

### Analysis

Open and axial coding was used for the thematic analysis. Information was broken down to common ideas (open coding), and information was grouped into overarching themes (axial coding) [6].

### Research ethics

The protocol was reviewed and approved by the University Health Network Research Ethics Board. All interviews were conducted voluntarily with informed consent, and the protection of confidentiality was always maintained.

## Results

Thirty-two patients were interviewed. The median age of participants was 52 (range 18–76). The median wait time for a patient from presentation with the symptom for which the MRI was ordered until they were first told about finding was 6.5 weeks (range 1–36 weeks). The level of satisfaction for the initial breaking of the news by the primary physician for patients ranged from 1 to 5 (on a 5-point scale, 5 being the best) with an average score of 4.1. All patients had structural lesions: 29 had a benign brain tumor, two had a cavernoma, and one had a chronic subdural hematoma.

## Thematic analysis

Analysis of the transcripts yielded four overarching themes, which are outlined below accompanied by some illustrative quotes.

1. A patient’s emotional status over the incidental finding is largely dependent on how they were informed of the news.
  Hearing the news that an MRI has shown an incidental finding is worrisome news. A family doctor usually breaks the news. Not surprisingly, patients were all very nervous and scared upon initially hearing the news. Most were told by their family physicians and liked how the news was relayed to them.
  “Our family doctor was perfect, he was very gentle and kind. He made me feel better about the whole situation.”
  The way a doctor explains the situation is also a big factor on whether the news causes panic or not.
  “It didn’t seem like a big deal; there was no concern or crisis expressed by the doctors. So I did everything in a relaxed way, I was pretty happy about it.”
  “The doctor didn’t want to tell me anything because she didn’t want to misdiagnose it; I waited a month and a half and I was so nervous my blood pressure went up.”
  Some patients also reported that the office staff also had a tremendous impact on how they viewed the whole situation.
  “I was really scared; the office was not calm about what they found. I was shown pictures of tumors even before I found out what I had. It was scary…”
2. Breaking worrisome news is best done in person, but if a patient has a good relationship with their doctor then telephone communication is also acceptable.
  The unspoken rule is that when delivering unexpected news, whether it is good news or bad, it should be done in person. This is the common expectation, and it was found in this study that most patients (84 %) were told the news of their incidental finding in person at an office visit. When asked if patients would prefer to be told in person or over the telephone in the comfort of their home, the majority (91 %) replied that they would rather go out of their way to an office visit and be told the news in person.
  “If the doctor tells you everything over the phone as they would in person then the phone call might be better even more convenient. But I think people just being people might prefer having someone make eye contact with you and talk to you.”
  “It’s impersonal over the phone; the doctor doesn’t know the physical and mental status of a person when they are telling them bad news. Its also hard to think of all the questions you want answered.”
  However, some of the patients that were told over the phone liked it. This has a lot to do with personal preferences. In the following patient testimony, it is seen that some patients do have a lot of anxiety problems, and just knowing that they have to go into a doctor’s office may make them even more stressed and nervous than getting the news right away would.
  “Normally phone isn’t good but in our case and for those who get very nervous, it was perfect because we knew right away. If we had to go into an office, the whole way there we would be stressed.”
  It would also depend upon the comfort and trust level that one has with her/his doctor. Those who are sure that they will be getting all of the information over the phone from their doctor said that they would not mind being told over the phone, especially patients who had occupations in the health field and knew more about MRI findings than an average person.
  “I am a well-informed patient, so over the phone wouldn’t be bad for me, but in general it would be hard to read a patient’s reaction over the phone. It also depends on what kind of relationship you have with your doctor.”
3. Patients are divided in their views about being informed of the possibility of an incidental finding being found on MRI
  Although some researchers have suggested it might be beneficial to inform patients of the chances of incidental findings on an MRI scan [13], through the study it was found that roughly half (52 %) would not like to be told this information prior to their scan. The most common reason patients gave reflected the old saying ‘ignorance is bliss.’
  “No knowledge is better, once you know for sure if you have a problem or not, then you can go about it.”
  Some also said that knowing about possibilities could be more of a hindrance than help, especially for those more prone to worrying.
  “Would it make any difference, knowing of the chances? It might make me more nervous. Better to wait for MRI results.”
  “Doctors should not inform about possibilities in advance to patients. It could create mental torture and more frustration with unknowns.”
  However, there was still a significant number of patients, roughly half (48 %) who said that they would have liked to have had been told about the chance of an incidental finding. The most common reason was so patients do not feel so surprised when they do get the news of a finding.
4. Waiting for the neurosurgical consultation is a stressful time without adequate support.
  After the family doctor has initially revealed the finding, patients have to wait to get a specialized opinion from a neurosurgeon. The wait time for patients to get a referral and an appointment with a neurosurgeon is very varied. For this study, it ranged from 1 week to 6 months, and every delay was due to delay in initiation of the referral (i.e., not on the part of the neurosurgeon). Physicians who do not have specialized training to give answers may refuse to talk further to the patient about the finding. This can be a very daunting time for patients, especially those without any answers and support.
  “It felt like a roller coaster. There’s a fear factor, a large quantity of unknown; it’s like being in the middle of a mental desert and there’s a sandstorm and you don’t know what to do.”
  Some patients experienced worsening of their original symptoms when they got the news of the finding.
  “When you forget little things you think the tumor is taking over; you try hard to not forget things and end up reinforcing it. My mind was always occupied with this.”
  “While I waited for the consult I started worrying more and more about the headaches I’d get, and it seemed to increase them.”
  Most patients would have liked to be told the news right away to lessen their worry.
  “The timing is critical; you need to tell patients right away; it’s like you are trapped inside your own head. Literally. It’s a caging feeling.”
  After the meeting with the neurosurgeon, all 32 patients said that they were immensely relieved that their questions got answered, to have an expert opinion on the subject, and of course to learn they did not have a life-threatening condition.

## Discussion

The results of this qualitative study show how patients have a wide variety of emotions from the time they first hear about their MRI findings up until they meet with a neurosurgeon to discuss the results. Although there have been many previous studies that looked at incidental findings, our study was the first to specifically examine the period between when the patient first hears of the discovery up until when they meet with the neurosurgeon, therefore giving a unique perspective on the patients’ anxieties and thoughts during this time. We found that how patients are informed about the findings by their family doctors has a large impact on how they are able to handle the situation and also how they cope during the wait time to see the neurosurgeon.

It is not only what physicians choose to say, but also how they say it that will really affect a patient. While breaking bad news is part of a physician’s job, less than a quarter have the proper training to do so [1]. A previous study had shown that even a physician’s posture while delivering the news can affect how the patient feels about the situation [3]. It is clear that physicians need to keep in mind how their tone and mannerisms affect the start of what will most likely be an emotional journey for their patients. Our study showed that those patients who were told in a calm and relaxing manner felt much better about the situation than those who were not. It was also found that patients understood that their family doctors did not have much in-depth knowledge of the subject of brain lesions, but those who found out that their doctors had done some research specifically for their case felt much happier and more informed with their doctors and with the finding. Even small changes in the physician’s tone can drastically affect the patient’s views of the issue, of their doctor, and most importantly whether or not they will follow through with the recommended medical route [4].

Most patients agree that face-to-face conversation is the ideal way to inform them of worrisome news. However, for some, a telephone conversation would also be acceptable. This is especially true for patients prone to high anxiety levels and those who have a close relationship with their physicians and can talk over the phone comfortably. In contrast, the majority of participants in a study by Schmidt and colleagues preferred to be sent a letter of their incidental finding right from the radiologist, with only a third of the participants requesting a one-on-one interview with the radiologist and only one preferring a phone call [12]. Perhaps patients should be asked about their communication preferences when first meeting a doctor so that both physician and patient are equally happy with the method of communicated practiced.

A clear finding in our study was that patients would like to be told exactly what their finding means as soon as possible. One patient even suggested that the radiologist should inform patients right away. While this is not a very realistic option, more should be done to provide support for patients during the time that they are waiting for the consultation. The wait times can range widely depending on how promptly the family physician initiates the referral to which neurosurgeon and whether the diagnosis is within the areas of interest of the neurosurgeon. When a surgeon is prioritizing her/his referrals for consultation, he/she may prioritize patients with “incidental findings” as very elective compared to more urgent patients, and this may add to the patient’s wait. As mentioned previously, our study was unique in that it looked at how patients felt during this time, and it showed that patients are especially vulnerable to an increase in nervousness and anxiety during this period. In some patients, an increase in symptoms was observed through their constant thoughts and uncertainties upon the matter, and one patient found that she could not hold a job during this time. Therefore, support needs to be available for these patients during the potentially long wait times.

A recent study by Drazin and colleagues suggested that patients are especially nervous about this meeting because of fears of a potential surgery and a lack of understanding of the medical terminology often used by neurosurgeons [5]. Neurosurgeons may not give a great amount of attention to patients that present with an incidental finding as it is generally not a serious health issue in comparison to most of the patients they treat. These patients may thus be prioritized lower than other patients and subsequently have longer wait times for consultation with the neurosurgeon. Perhaps with more attention and awareness of the anxiety the patient, neurosurgeons may be able to improve wait times for these patients. We humbly suggest that neurosurgeons should consider prioritizing patients referred for “incidental findings” more urgently, as a brief consultation can save the patient and the referring doctor weeks or months of undue anxiety.

Family physicians should consider having regular checkup meetings with patients to see how they are coping until the diagnosis is made clear by a neurosurgeon and also a follow-up with them after the diagnosis to answer any lingering questions patients may have. This can be especially helpful due to the fact that in our study all of the patients found that the initial symptoms that led to the MRI scan were found to be not a result of the brain lesion that resulted. These lingering symptoms can be followed up further with the family physician.

### Limitations

There are a few limitations to our study. First and foremost is that qualitative research methodology is foreign to many clinicians, and especially neurosurgeons, and may be difficult to digest, but it is the only methodology available to answer certain questions. Second, all of the patients interviewed were from one hospital (Toronto Western) and saw one neurosurgeon specializing in brain tumors. Therefore, this study may not be applicable to all patients with incidental findings and those at other hospitals or in different cultures. Finally, the interviews took place in a hospital setting after the patients met with the neurosurgeon, so patients might have felt some internal pressure to speak favorably of their experience. This was reduced as much as possible by the interviewer having no relationship with the patient, by asking open-ended questions and encouraging patients to expand on their answers and feelings as freely as possible, and by the interviewer emphasizing the patients’ anonymity.

## Conclusion

The findings obtained in this study provide some recognition of how patients feel after getting the news of an unexpected finding on their brain imaging. Patients are understandably nervous and afraid during this time, but our study also showed how the experience could be made more comfortable for patients right from the start, from being told the news in a calm and sympathetic manner, to providing support for patients while they wait for a referral meeting with a neurosurgeon, to expediting that consultation. Overall, patients are generally happy with the experience and the care that they receive, but little changes in the health care system could be easily implemented to make this experience better.

## Interview Guide

Read to participant:

This study is examining the views of patients and physicians on breaking bad news about incidental findings on MRI scans. During this interview, I will be asking you questions about how you felt during the whole process from the initial finding up until today’s consult with the neurosurgeon. If you have any questions, please feel free to ask; if any question needs to be repeated or rephrased, just let me know. Remember that this is completely voluntary, and your time and answers are much appreciated; however, if you happen to feel uncomfortable with any question you are welcome to skip the question and also to end the interview at any time. Do you have any questions?

1. Why did you go for a brain scan? Who sent you?
2. Who told you about the findings? Was it by telephone, in person, or email?
3. Which method would you have preferred?
4. How did you feel when you first found out?
5. Did you feel satisfied with the information you got from the primary source?
6. How long did you wait for a consult with the neurosurgeon?
7. During this time, how were you feeling both emotionally and physically?
8. What are your thoughts or issues about the results now?

Case Scenarios

1. You have gone for an MRI scan because of a headache you have been having. The results come back and your doctor calls you and tells you that they have found something and you need to go have a consult with a neurosurgeon. Do you think this is an appropriate way of breaking the news? Why or why not? Please explain.
2. Prior to your MRI scan, you do some research and realize that incidental findings are very common in the general population. You were not told this by your doctor who had sent you for the MRI. Would you have preferred to know about this prior to getting the MRI? Please explain.
